# Influence of Deep-Frying Temperature on the Flavor Profile and Quality Attributes of Batter-Coated Chicken Strips: An Integrated GC–IMS and E-Nose Study

**DOI:** 10.3390/foods15213860

**Published:** 2026-10-28

**Authors:** Xiaoping Feng, Ting Bai, Lu Zhang, Zhongyan Zhu, Shuangli Xiong, Chengjian Xu, Anlin Li, Ling Hua, Xinyi Wu

**Affiliations:** 1 Food Nutrition and Health Key Laboratory of Sichuan Universities, Sichuan Tourism University, Chengdu 610100, China; 2 College of Culinary and Food Science Engineering, Sichuan Tourism University, Chengdu 610100, China; 3 College of Sichuan Provincial Key Laboratory of Meat Processing, Chengdu University, Chengdu 610106, China

**Keywords:** batter-coated, chicken breast strips, deep-frying temperature, volatile compounds, gas chromatography–ion mobility spectrometry, E-nose

## Abstract

This study investigated the influence of deep-frying temperature (150, 160, 170, 180, and 190 °C, each for 170 s) on the flavor profile and quality attributes of batter-coated chicken breast strips using integrated electronic nose (E-nose) and gas chromatography–ion mobility spectrometry (GC-IMS) analyses. The results demonstrated that frying temperature significantly modulated the formation of volatile compounds and overall product quality. The optimal sensory score (83.0 ± 0.82) and the most desirable flavor characteristics were achieved at 170 °C, where oil content (wet basis, whole product) peaked at 49.8% and then declined at higher temperatures. A total of 60 volatile compounds were tentatively identified across all samples. Through variable importance in projection (VIP > 1) screening, 10 key differential volatile compounds were identified, including four alcohols, three aldehydes, one acid, one pyrazine, and one alkane. Correlation analysis revealed that cinnamyl alcohol, 2-methylpyrazine, and butyraldehyde-D were positively correlated with sensory attributes, whereas 3-methylbutan-1-ol exhibited a negative correlation. Odor activity value analysis further identified hexanal-M, butyraldehyde-M, and 3-methylbutan-1-ol-M as the predominant aroma-active compounds across all temperatures. Overall, these results indicate that frying temperature is a key factor influencing the balance between lipid oxidation and Maillard reaction pathways, thereby affecting the volatile profile and sensory quality of battered fried chicken products. The combined E-nose and GC–IMS approach appears to be effective for characterizing flavor dynamics during deep frying and could offer valuable insights for process optimization in the fried chicken industry.

## 1. Introduction

Deep-fat frying, typically conducted at 140–190 °C, is a widely used culinary technique that produces foods with a desirable crispy texture, golden-brown color, and unique aroma [1,2]. However, the high oil content of fried foods poses health concerns, as excessive consumption is linked to obesity, cardiovascular diseases, and hypercholesterolemia [3]. With increasing consumer demand for healthier, low-fat foods [4], maintaining the unique aroma of fried foods while reducing their oil content has become a pressing concern for consumers and a critical scientific issue to address.

To mitigate oil uptake, various strategies have been explored, including the optimization of frying parameters and the application of batter coatings [4]. A batter coating serves as a physical barrier and structural matrix; during frying, starch gelatinization and protein denaturation form a porous crust that modulates moisture–oil exchange. A well-formulated coating that promotes rapid crust formation can effectively limit oil ingress [5]. The role of the coating and the frying temperature are critically interdependent. For instance, Barbut (2013) compared skinless chicken breast fillets fried at 190 °C with and without a batter coating [6]. Non-coated samples developed a dry, brittle crust with substantial weight loss and muscle damage, whereas coated products maintained structural integrity, a golden-brown appearance, and minimal oil uptake. Liu et al. (2024) systematically investigated the effects of frying temperature (140–180 °C) and time on batter-coated meat products, reporting that moisture content decreased with increasing temperature, while oil content first increased and then decreased [7]. These findings underscore that both the coating and the frying temperature critically determine the final quality of fried meat products.

Among the controllable process variables, deep-frying temperature is an important factor, as it directly dictates the rate of key physicochemical reactions—including starch gelatinization, protein denaturation, and the Maillard reaction—which collectively determine the final product quality [8]. The central hypothesis of this study is that frying temperature will exert a non-linear and multi-faceted influence on batter-coated chicken strips. Specifically, low temperatures may result in inadequate crust formation, excessive oil absorption, and a pale, underdeveloped flavor. Conversely, excessively high temperatures may lead to a hard, dry texture, over-browning, and the generation of undesirable burnt or off-flavors. The optimal temperature is expected to balance these competing effects, yielding a product with superior sensory qualities and a more desirable flavor profile.

To comprehensively evaluate the aroma characteristics of fried foods, advanced analytical techniques are indispensable. The electronic nose (E-nose) is a rapid, non-destructive tool for discriminating overall flavor profiles but lacks the specificity to identify individual volatile compounds. In contrast, targeted approaches such as gas chromatography-olfactometry-mass spectrometry combined with Odor activity value (OAV) analysis are required to identify key aroma-active compounds in chicken matrices [9]. Gas chromatography–ion mobility spectrometry (GC-IMS) is a powerful technique that synergistically combines the high separation capacity of GC with the rapid response and high sensitivity of IMS. It is particularly effective for analyzing complex volatile mixtures in food matrices, enabling a detailed understanding of the formation pathways of key aroma-active compounds.

Accordingly, the objective of this study is to systematically investigate the influence of deep-frying temperature (150–190 °C) on the quality attributes of batter-coated chicken breast strips. A multi-analytical approach integrating sensory evaluation, texture analysis, oil content measurement, an E-nose, and GC-IMS was employed. Multivariate statistical methods, including principal component analysis (PCA) and orthogonal partial least squares-discriminant analysis (OPLS-DA), were applied to decipher the relationships between frying temperature, volatile compound profiles, and sensory acceptability. This study aims to identify the optimal frying temperature that maximizes sensory quality while minimizing oil content and to elucidate the underlying chemical mechanisms governing flavor formation, thereby providing a theoretical basis for precision processing of high-quality fried chicken. While the Introduction highlights health concerns associated with high oil content, the present study does not evaluate low-fat pretreatments; rather, it examines how frying temperature alone governs the balance between oil uptake, texture, and flavor, thereby providing a basis for subsequent low-fat strategy development.

## 2. Materials and Methods

### 2.1. Materials

The frozen chicken breast was sourced from Shandong Yashixiang Meat Food Co., Ltd., Jinan, Shandong, China. The raw material was commercial White Feather broiler breast; birds were slaughtered at 35–42 days of age, and carcasses were chilled at 0–4 °C for post-mortem aging, trimmed and portioned, then blast-frozen and stored at −18 °C throughout the cold chain. It was thawed with running water for 2 h and cut into strips measuring approximately 5 cm in length, 1 cm in width, and 1 cm in height. The thawing endpoint was judged by the absence of a hard frozen core; all temperature groups used the same batch of raw material and identical thawing protocol, so any thawing-related variation in drip loss or water-holding capacity would be consistent across groups and would not confound the temperature comparison. The surface water on the chicken breast strips was absorbed with absorbent paper.

### 2.2. Process for Deep-Frying Batter-Coated Chicken Breast Strips

Five mL of soy sauce and 4 mL of oyster sauce were added to 100 g of chicken breast strips. The batter was formulated with a fixed proportion of raw materials: 20% wheat flour, 16.4% sweet potato starch, 4% kudzu root starch, 0.6% baking starch, and 59% water. This batter formulation was adapted from our previous optimization study on battered fried meat [10], in which these proportions were optimized by response surface–principal component analysis. After thorough mixing, the batter was stored at 4 °C for 2 h and equilibrated to room temperature (approximately 20–25 °C) before dipping. The chicken strips were then immersed in the batter for 10 s, slowly lifted out, and allowed to drip for 2–5 s to remove excess batter. Coated strips were deep-fried in grade-3 rapeseed oil (GB/T 1536, Daodaoquan Grain and Oil Yueyang Co., Ltd., Yueyang, Hunan, China) [11] with continuous flipping to ensure uniform heat distribution. Fresh oil was used for each frying batch, and the oil was discarded after each temperature group; thus, each of the three biological replicates at each temperature was fried in fresh oil. Although the smoke point of grade-3 rapeseed oil is relatively low, the short frying time (170 s) limited oil degradation, and the use of fresh oil for every batch eliminated oil deterioration as a confounding factor across temperature groups. Five frying temperature levels were set at 150, 160, 170, 180, and 190 °C [2], with a consistent frying duration of 170 s for all treatments. All groups used an identical batter formulation and standardized batter preparation, dipping, and draining procedures, so that frying temperature was the primary variable. Batter viscosity was not separately measured for this batch, and coating pick-up was not quantitatively verified; because the same batter batch and identical dipping (10 s) and draining (2–5 s) procedures were applied to all groups, any variation in these parameters would be random rather than systematically associated with temperature. After frying, samples were cooled to room temperature prior to further measurements, with three biological replicates per group.

### 2.3. Sensory Analysis

For sensory analysis, a quantitative descriptive analysis was conducted. A sensory evaluation team consisting of 10 individuals (aged 18–25 years, 1:1 male-to-female ratio) evaluated each sample and rinsed their mouths with water. After 2 min, the next sample was evaluated. The scoring criteria were as described in our previous research [10], and the indices are not shown. The sensory rating was based on a 100-point scale, including 20 points for color, 25 points for appearance, 30 points for odor, and 25 points for taste.

### 2.4. Texture

The crust and meat core of rectangular crispy fried chicken breast strips were separated before testing. Measurements were taken at the top, middle, and bottom of each sample to reduce structural deviation. TPA was conducted using an R25 probe mounted on a TMS-pro texture analyzer (Food Technology Corporation, Sterling, VA, USA) with slight adjustments to a standard two-compression procedure [12]. Test parameters: speed 60 mm/min, compression strain 70%, trigger force 0.375 N, and interval dwell time 2 s. Hardness, springiness, gumminess, chewiness, and resilience were determined. Each group was measured in quintuplicate, and results were averaged. Sample dimensions were approximately 5 cm × 1 cm × 1 cm, consistent with those used for frying.

### 2.5. Oil Content

The oil content is defined as total fat content on a wet basis for the whole product, including the batter coating and core. The measured values therefore comprise the oil absorbed during frying, the intrinsic fat of the chicken breast, and the fat contributed by the batter ingredients. Intrinsic fat was not subtracted, and results are not expressed on a dry basis. The fried and batter-coated chicken breast strips were minced using a meat grinder until a homogeneous, paste-like matrix was obtained; no further sieving was applied, and particle size was not measured. The oil content was determined by solvent extraction with petroleum ether following the Soxhlet principle, using a fully automatic fat analyzer (SOX606, Shandong Haining Scientific Instrument Co., Ltd., Jinan, Shandong, China). The oil content was calculated according to the following formula [10]:

$$X=\frac{m_{1}-m_{0}}{m}\times 100\%$$

where *X* is the oil content (g/100 g), m_1_ is the mass of the solvent cup with oil (g), m_0_ is the mass of the dried solvent cup (g), and m is the sample mass (g).

### 2.6. E-Nose

The fried chicken breast meat samples with consistent deep-frying conditions, sizes, and shell wrapping statuses were uniformly crushed, followed by screening array fingerprint analysis (Shanghai Baosheng Industrial Development Co., Ltd., Shanghai, China). The sensors and corresponding performance of the E-nose are shown in Table 1. The E-nose system consisted of a sampling device, a detector unit comprising six metal oxide sensors, and pattern recognition software for data recording and analysis. E-nose analysis was performed according to a previous method with slight modifications [13]. In brief, 5 g of the sample was weighed and placed in a 40 mL sample bottle, which was sealed with a lid and left to stand at 25 ± 2 °C for 60 min to stabilize the gas composition at the top. After that, headspace sampling was used for determination. Seven replicates were conducted for each sample. The internal gas flow rate was 1 L/min, the sensor cleaning time was 100 s, and the sample testing period was 150 s. The E-nose output was recorded as sensor response intensity (instrumental response units) and used directly for radar plotting and PCA without further transformation.

### 2.7. GC–IMS

The volatile compounds were measured via gas chromatography ion mobility chromatography (Germany G. A. S. Company). The sample was put into a 20 mL headspace vial and incubated at 60 °C for 15 min before injection. The MXT-WAX chromatographic column measured 30 m in length, with an inner diameter of 0.53 mm and a film thickness of 1.0 μm. The temperature of the column was 60 °C. The detection time was thirty-five minutes. The flow rate program for the carrier gas, which was N_2_ (purity ≥ 99.999%), ran as follows: 2 mL/min at first, maintained for 2 min, then gradually increased to 20 mL/min in 8 min, then maintained at 100 mL/min for 10 min, and finally linearly increased to 150 mL/min for the final 10 min. The IMS temperature was 45 °C, and the flow rate was 150 mL/min. With an injection volume of 300 μL, an incubation duration of 15 min, an incubation temperature of 60 °C, an injection needle temperature of 85 °C, an incubation speed of 500 r/min, a linear voltage of 500 V/cm within the drift tube, and a drift tube length of 5.3 cm, the system was an automatic headspace sampling device. OAV was calculated as the ratio of relative content (%) to odor threshold (mg/kg) according to Nie et al. (2024) [9]. OAV > 1 indicates a likely aroma contributor. For every sample, three replicates were made.

### 2.8. Statistical Analysis

Microsoft Office 2021 (Microsoft Corporation, Redmond, WA, USA) was used to analyze the data. SIMCA 14.1 (Umetrics, Umea, Sweden) was applied to perform the PCA and orthogonal partial least squares discriminant analysis. Peak volume histograms of volatile aroma compounds and E-nose radar graphs were drawn via Origin 2021 (OriginLab Corporation, Northampton, MA, USA). The volatile chemicals were analyzed informally on the basis of retention and drift times via the VOCal software v0.2.1 (G.A.S. Gesellschaft für Analytische Sensorsysteme mbH, Dortmund, Germany) embedded in the GC–IMS system. The GC–IMS system’s built-in reporter plug-in was used to instantly produce two-dimensional spectra of volatile aroma compounds, while the gallery plot plug-in generated their fingerprint profiles. Data were collected from three parallel experiments with different samples, and the average was calculated. The results are presented as the mean ± standard deviation (SD). One-way ANOVA with Duncan’s post hoc test (SPSS 21.0, IBM Corporation, Armonk, NY, USA) was used to compare sensor responses among frying temperatures (*n* = 7 parallel measurements per temperature), with significance set at *p* < 0.05. Significant differences in the data were assessed using the Duncan test in SPSS 21.0 software, with significance determined at *p* < 0.05. Figures were created using Origin 2021 software (Origin Lab, Inc., Northampton, MA, USA).

## 3. Results and Discussion

To comprehensively evaluate the quality attributes of batter-coated chicken strips as a function of frying temperature, a multi-dimensional analytical strategy was employed, encompassing sensory evaluation, instrumental texture profiling, physicochemical property measurements (moisture and oil content), and volatile flavor analysis (E-nose and GC-IMS). This integrated approach allowed for a systematic investigation of the temperature-dependent changes in crust formation, texture, oil uptake, and flavor development.

### 3.1. Sensory Scores and Texture

**Figure 1:**
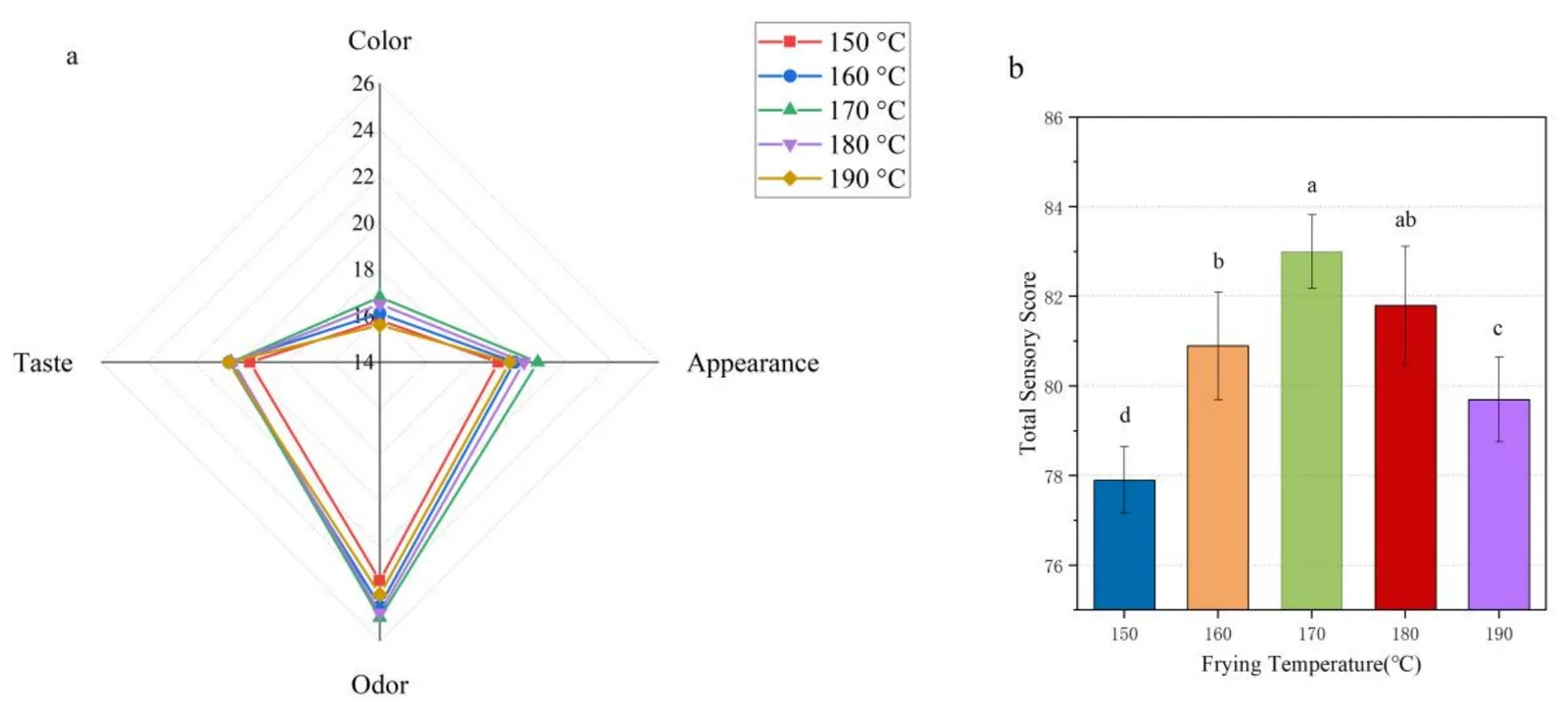
Sensory attributes and total sensory scores of batter-coated chicken strips fried at different temperatures. (**a**) Radar plot of sensory attributes of batter-coated chicken breast strips fried at different temperatures; (**b**) Total sensory scores of batter-coated chicken breast strips fried at different temperatures. Different lowercase letters indicate significant differences at *p* < 0.05 according to Duncan’s test.

Frying temperature significantly modulated product quality by regulating starch gelatinization, protein denaturation, and thermal browning reactions. As shown in Figure 1, sensory scores increased initially and then declined, with the maximum total sensory score (83.0 ± 0.82) obtained at 170 °C. Low-temperature frying (150–160 °C) caused an insufficient Maillard reaction, leading to pale color, incomplete batter shaping, and weak fried flavor. Excessively high temperatures (180–190 °C) induced over-browning and a slight burnt odor, deteriorating sensory acceptability.

As shown in Table 2, texture analysis provided instrumental support for sensory perceptions of crispness and mouthfeel. Hardness and chewiness increased steadily with temperature, but sensory crispness was optimal only at moderate hardness. At 150–160 °C, low hardness (11.22–13.00 N) and chewiness (2.40–2.53 mJ) corresponded to a soft, soggy crust with insufficient crispness and greasiness. At 180–190 °C, excessive hardness (30.70–35.31 N) and chewiness (4.65–4.74 mJ) produced a hard, tough texture that compromised palatability. Only at 170 °C did the moderate hardness (18.20 N) and chewiness (2.72 mJ) yield a crispy crust and tender interior, achieving the highest sensory scores for taste and odor. Thus, 170 °C represented the optimal balance where crust formation, oil uptake, and flavor development converged for maximum consumer acceptability. Instrumental hardness and chewiness increased monotonically with temperature, whereas the total sensory score peaked at 170 °C and declined thereafter. This divergence indicates that instrumental texture alone cannot predict sensory acceptability: beyond 170 °C, excessive crust hardening outweighed any gain in springiness and reduced overall palatability.

### 3.2. Oil Content Analysis

**Figure 2:**
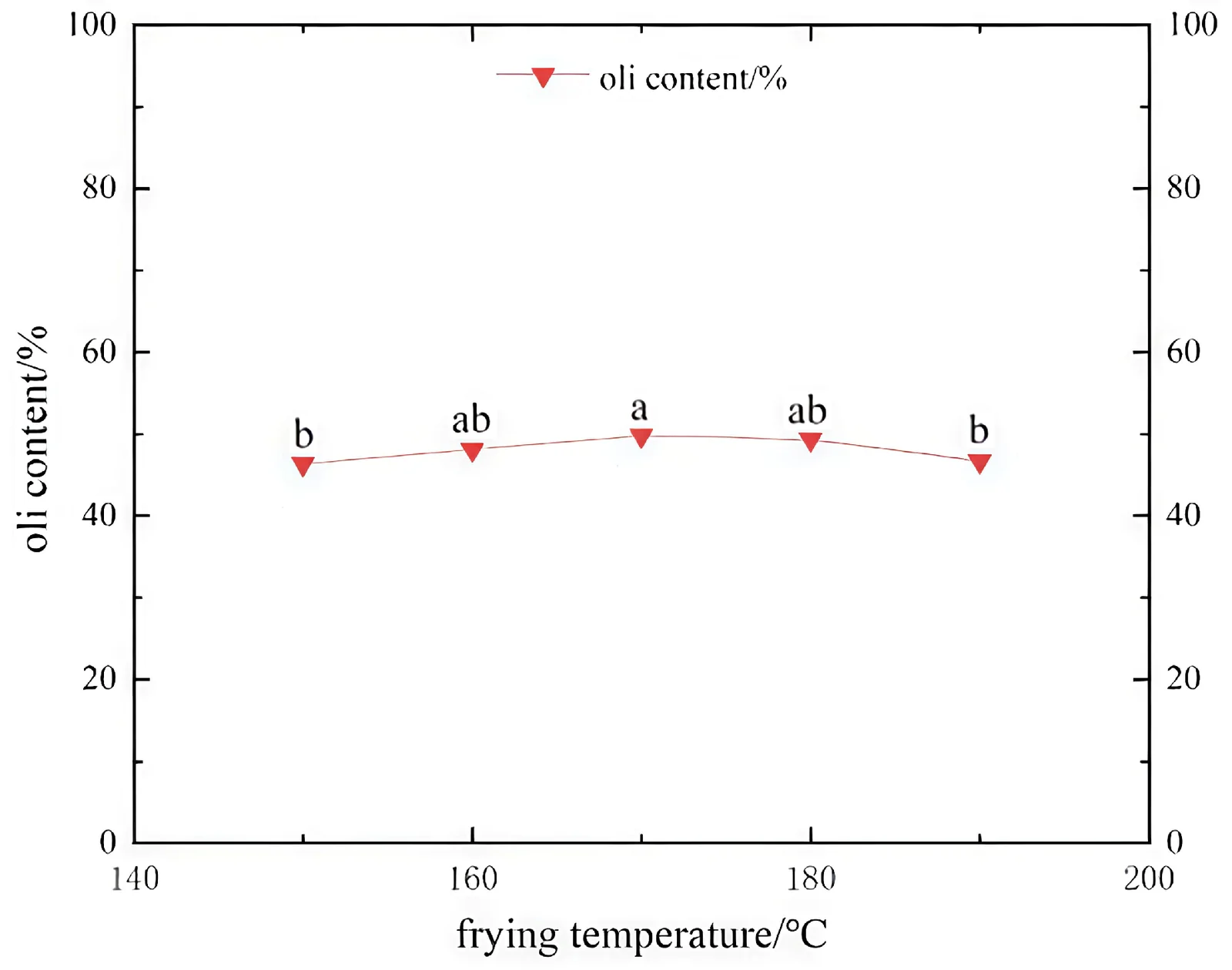
Effects of different temperatures on the oil content of deep-fried chicken breast strips. Different lowercase letters indicate significant differences at *p* < 0.05 according to Duncan’s test.

As shown in Figure 2, the oil content of batter-coated chicken strips increased first and then decreased with rising frying temperature, peaking at 170 °C. Oil absorption is a key characteristic of fried battered products, governed by moisture–oil exchange during heating. The maximum oil content reached 49.8% at 170 °C, which was statistically comparable to samples fried at 160 °C and 180 °C (*p* > 0.05), yet significantly higher than those treated at 150 °C and 190 °C (*p* < 0.05). These value were higher than oil content values commonly reported for breaded chicken products (approximately 18–25%), primarily because it represents total fat in the whole product including the coating, on a wet basis, whereas many literature values refer to the core only or a dry basis. Within a moderate temperature range, accelerated water evaporation generates porous pathways in the batter, facilitating oil uptake via water-oil replacement [5]. Excessively high temperatures induce rapid starch gelatinization at the batter surface, forming a compact crust that blocks oil penetration and reduces the final product’s oil content. Collectively, 170 °C was the optimal frying condition for battered chicken strips. This temperature achieved a superior balance of sensory performance and textural properties, characterized by desirable crispness, low greasiness, and the highest overall palatability and acceptability.

### 3.3. E-Nose Analysis

**Figure 3:**
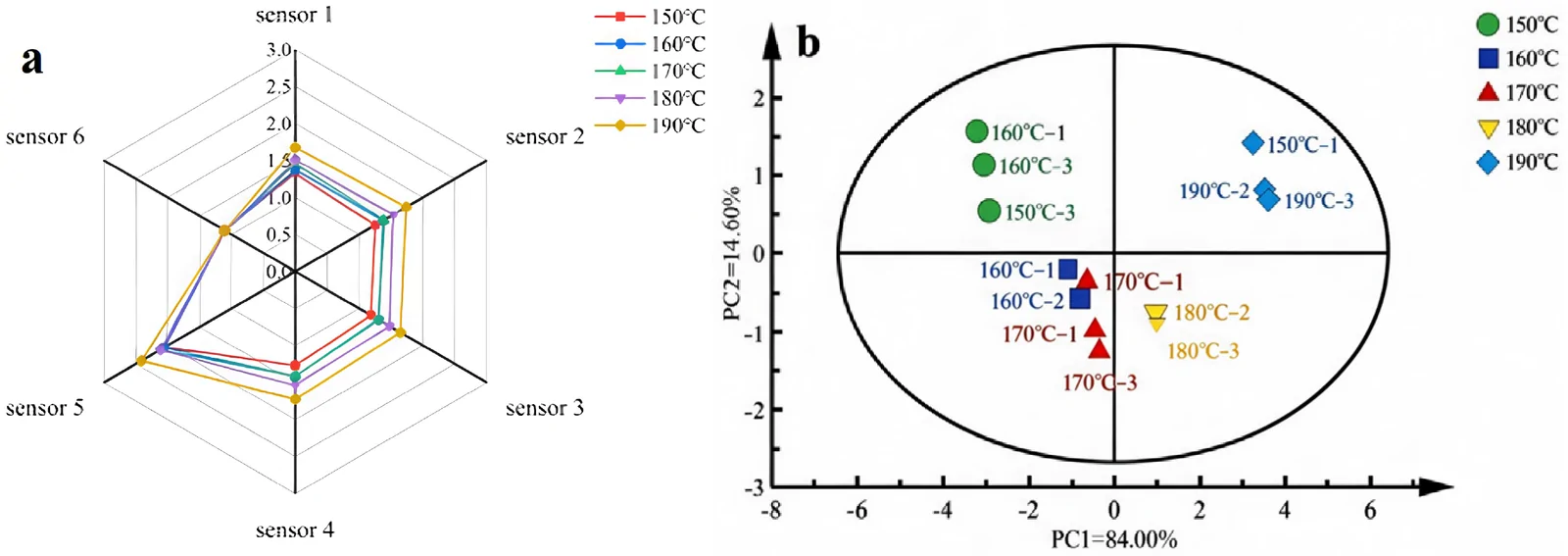
E-nose data analysis of the effects of different deep-frying temperatures on volatile aroma compounds in battered chicken breast strips (radar plots (**a**) and PCA plots (**b**)). Values in (**a**) represent sensor response intensities (instrumental units). For clarity, significance letters are not shown in the radar plots; the statistical comparisons among temperatures are reported in Section 3.3.

The E-nose response profiles of deep-fried battered chicken breast strips differed among frying temperatures (Figure 3a). For clarity, significance letters are not shown in the radar plots, and the statistical results are reported in the text below. One-way ANOVA with Duncan’s post hoc test (*n* = 7 parallel measurements per temperature) showed that the responses of sensors S2, S3, and S4 increased significantly with rising frying temperature (*p* < 0.001). For S2 and S4, the response at 150 °C was significantly lower than at 160–190 °C, with the highest values observed at 180–190 °C. S3 showed a similar temperature-dependent increase, with 180–190 °C significantly higher than 150–170 °C. In contrast, the response of S6 showed no significant temperature dependence (*p* = 0.23), consistent with the overlapping S6 traces in the radar plot. S1 and S5 showed overall significant differences among temperatures (*p* = 0.020 and *p* = 0.039, respectively), although pairwise separation was limited by within-group variance as indicated by the letters Figure 3a). The highest absolute responses were observed for S5, followed by S2, S4, S3, S1, and S6.

The overall aroma patterns of the five sample groups were further analyzed by PCA (Figure 3b). The first two principal components accounted for 98.6% of the total variance (PC1 = 84.0%, PC2 = 14.6%), indicating that the PCA model adequately captured the fragrance characteristics of the five groups. The suffixes −1, −2, and −3 denote the three biological replicates at each frying temperature. The contribution of PC1 was greater than that of PC2, and the further the samples were from PC1, the more distinct the aroma differences between the samples were [14]. The 150 °C, 180 °C, and 190 °C samples were located in the first, second, and fourth quadrants, respectively, and were clearly separated from the other groups [15], indicating significant differences in overall aroma among these samples. The E-nose could discriminate the overall smell characteristics of the samples but could not resolve subtle differences in individual aroma components; therefore, specific flavor differences were further analyzed by GC-IMS.

### 3.4. GC–IMS Analysis

#### 3.4.1. GC–IMS Spectral Analysis and Color-Canceled Comparison

**Figure 4:**
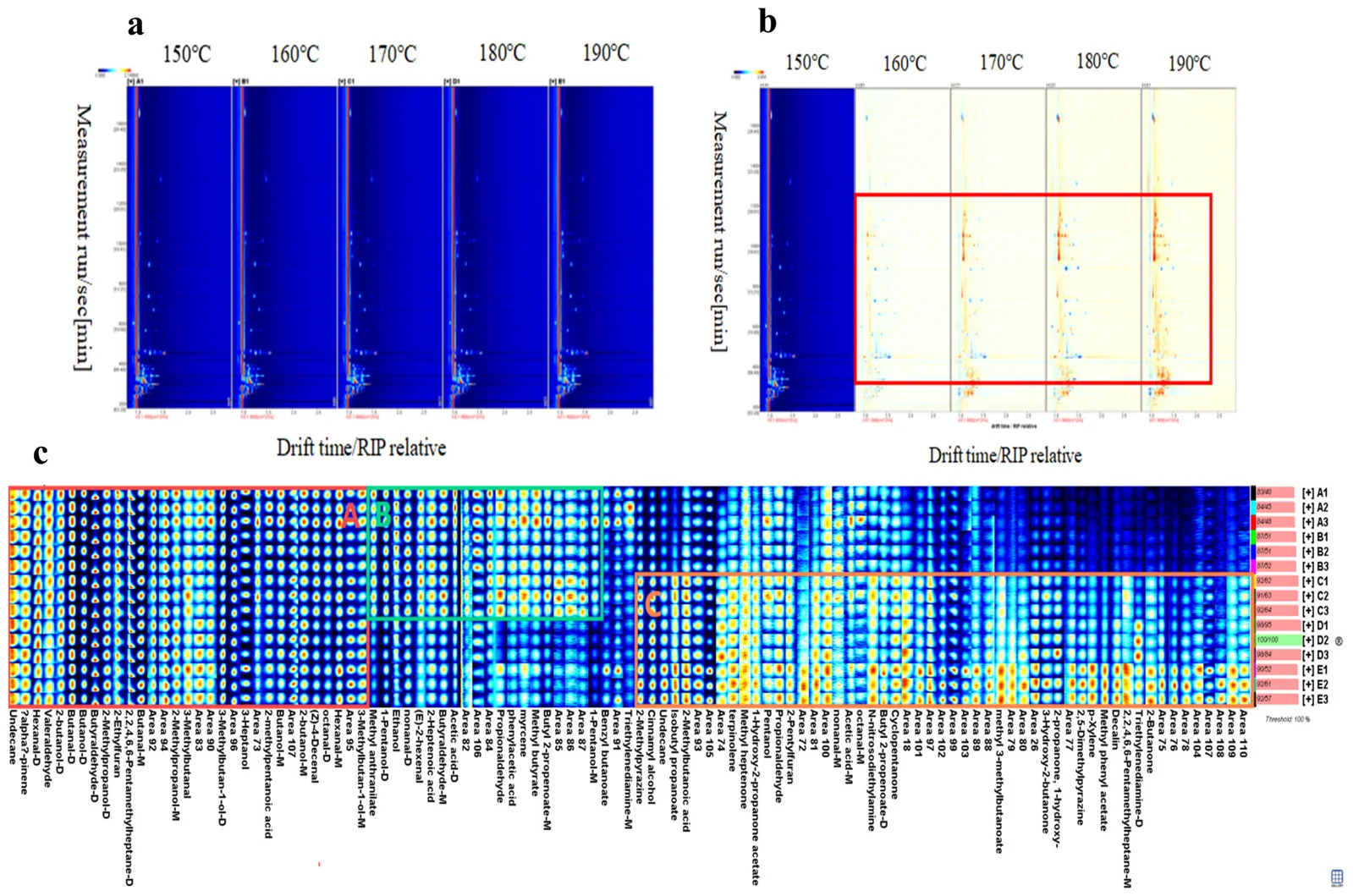
GC–IMS data analysis of the effects of different deep-frying temperatures on volatile aroma compounds in battered chicken breast strips (two-dimensional maps (**a**), difference spectra of volatile compounds (**b**), and fingerprint (**c**)). In panel (**b**), the red box highlights the region with obvious signal variations of volatile compounds at temperatures from 170 °C to 190 °C. In panel (**c**), the colored boxes A, B and C denote three characteristic regions with distinct volatile compound changing trends under different frying temperatures.

In the GC–IMS spectrum, the reaction ion peak (RIP) represents the reactant ion signal, and analyte-related signals appear separately from the RIP [16]. Points on both sides of the RIP correspond to aromatic substances, with color depth and spot area reflecting their relative signal intensity. Signal intensity increased with rising deep-frying temperature (Figure 4a), consistent with previous findings [17]. To better compare flavor differences among the five groups, the spectra of samples other than the 150 °C reference were color-canceled. Red indicates higher concentration than the reference, while blue indicates lower concentration [18]. As shown in Figure 4b, the relative content of flavor compounds gradually increased with rising deep-frying temperature.

#### 3.4.2. Fingerprint Analysis of Volatile Compounds

GC-IMS differential spectra showed no obvious differences in aroma compounds across samples. Therefore, fingerprint spectra were generated using the gallery plot plug-in, allowing intuitive visualization of individual volatile compounds (Figure 4c). Spot color intensity indicates relative signal strength, shifting from blue (low) to red (high); these colors represent relative signal intensity rather than calibrated absolute concentration [19]. The fingerprints showed good repeatability within groups and clear differences between groups. The apparent discrepancy between the lack of obvious differences in the differential spectra and the clear group separation in the fingerprint plots arises because the differential spectra display overall signal patterns at a single comparison level, whereas the gallery plot resolves individual compound signals across all samples simultaneously, making compound-specific differences visible.

The gallery plots were segmented into three sections (areas A, B, and C in Figure 4c). Notably, in area A, volatile compounds, including 3-methylbutan-1-ol-M, hexanal-M, (Z)-4-decenal, 2-butanol-M, butanol-M, 2-methylpentanoic acid, 3-heptanol, 3-methylbutan-1-ol-D, 3-methylbutanal, 2-methylpropanol-M, butanal-M, 2,2,4,6,6-pentamethylheptane-D, 2-ethylfuran, 2-methylpropanol-D, butyraldehyde-D, butanol-D, butanal-D, 2-butanol-D, valeraldehyde, hexanal-D, alpha-pinene, and undecane, did not change significantly with increasing deep-frying temperature. In area B, the contents of volatile compounds were significantly reduced when the deep-frying temperature was greater than 170 °C. These compounds included triethylenediamine-M, benzyl butanoate, 1-pentanol-M, butyl 2-propenoate-M, methyl butyrate, myrcene, phenylacetic acid, propionaldehyde, acetic acid-D, butyraldehyde-M, 2-heptenoic acid, (E)-2-hexenal, nonanal-D, ethanol, 1-pentanol-D, and methyl anthranilate, which subsequently lightened the color. A peak putatively annotated as triethylenediamine was detected. However, triethylenediamine is primarily known as an industrial catalyst for polyurethane foams and is listed as a potential migrant from food-contact materials. Given the absence of authentic standard confirmation and its known industrial origin, this compound is not considered a frying-derived flavor contributor and is not discussed further. In area C, at temperatures above 170 °C, contents of compounds such as 2,5-dimethylpyrazine, 3-hydroxy-2-butanone, cinnamyl alcohol, and 2-methylpyrazine increased significantly. These trends are broadly consistent with previous reports that higher frying temperatures promote Maillard-derived pyrazines and lipid oxidation products [17,20]. However, some discrepancies were observed. Specifically, the decrease in hexanal-M content above 170 °C contrasts with the continuous increase reported by Domínguez et al. (2014) in fried meat, possibly due to differences in batter composition and crust formation that limit oil penetration [21]. Additionally, the marked reduction in esters (e.g., methyl butyrate) at higher temperatures has not been widely documented, suggesting matrix-specific thermal degradation. These differences highlight the influence of the food matrix and processing conditions on volatile profiles and warrant further investigation.

#### 3.4.3. Characterization of Volatile Compounds

A total of 60 volatile compounds were tentatively identified in battered chicken breast strips fried at different temperatures, and the detailed information, including compound classification, is summarized in Table 3. These compounds consisted of 14 aldehydes, 14 alcohols, eight esters, eight hydrocarbons, five ketones, five acids, two pyrazines, two furans, and one amine (triethylenediamine, excluded from flavor discussion as noted above) [22,23].

Aldehydes, which are derived mainly from lipid oxidation, have low detection thresholds and play a significant role in meat flavor. Their total content increased with rising frying temperature, consistent with Fu et al. (2022), who reported that higher temperatures accelerate oxidative degradation of unsaturated fatty acids. However, individual aldehydes exhibited distinct behaviors [22]. For example, hexanal content gradually decreased with increasing deep-frying temperature, which may be explained by the rapid formation of a dense, crispy batter shell at higher temperatures that limits oil penetration and subsequent hexanal production. In contrast, butyraldehyde, a key oxidation product of fat, increased markedly with temperature. Similar to our findings, a recent E-nose/GC-IMS study on fried chicken fillets identified hexanal, nonanal, and butyraldehyde as key volatile flavor compounds, with butyraldehyde making a high contribution to the overall flavor across different frying oils [24].

Hydrocarbons, derived mainly from thermal degradation of fats or oxidative degradation of long-chain fatty acids, also increased with temperature, likely due to the combined effects of lipid oxidation, protein breakdown, and Maillard reactions [25]. Terpenes such as α-pinene, myrcene, and terpinolene, which impart pine and fruity aromas, were primarily derived from spices added during processing rather than from the chicken meat itself. As reported by Du et al. (2024), terpene contents initially increased with heating temperature and then decreased, reflecting the thermal lability of certain terpenoid compounds [26].

The values are the means ± SDs of triplicate samples. Means followed by different superscripts within the same column are significantly different (*p* < 0.05). The peak putatively annotated as triethylenediamine is listed for data completeness; however, given its known industrial origin and the absence of authentic standard confirmation, it was excluded from the flavor interpretation in this study (see Section 3.4.2).

Alcohols in the fried batter-coated chicken strips are unlikely to originate predominantly from microbial metabolism, given that the product is subjected to deep-frying at 150–190 °C. Instead, their formation is more plausibly attributed to three thermally relevant routes, with the dominant route depending on the individual compound. First, lipid oxidation generates aldehydes that can be subsequently reduced to the corresponding alcohols; this route likely accounts for 1-pentanol, butanol, 2-butanol, and 2-methylpropanol [27]. Second, 3-methylbutan-1-ol is classically formed via the Ehrlich pathway from leucine, but in a high-temperature frying system it can also arise from thermal Strecker degradation of leucine followed by reduction in the resulting 3-methylbutanal [28]. Third, cinnamyl alcohol, owing to its high boiling point (~250 °C), is relatively resistant to thermal decomposition and may therefore become relatively enriched at higher temperatures through retention rather than de novo formation. Except for the increase in cinnamyl alcohol content, the overall alcohol content decreased. This decrease may reflect the combined effects of thermal degradation, esterification, and condensation of alcohols at elevated temperatures, as well as the formation of a dense crust that can retain some volatiles within the product matrix while others are thermally decomposed [29].

Ketones, derived from lipid oxidation and amino acid degradation, have higher sensory thresholds than aldehydes but possess unique fruity and other fragrances. However, ketones do not significantly enhance chicken strip aroma, and excessive production is detrimental to human health. With increasing frying temperature, ketone content increases, likely due to condensation reactions between fatty acids and sugars, as well as thermal peroxidation of oils [30]. Therefore, controlling a reasonable frying temperature is particularly important for food safety.

Acids originating from aldehyde oxidation and enzymatic lipolysis have low thresholds, with short-chain acids playing a significant role in meat aroma [31]. Acetic acid accounts for most acid volatiles and is the primary source of sour aroma in chicken breast strips.

Esters, formed by esterification of carboxylic acids and alcohols [32], exhibit subtle fatty odors (long-chain) or fruity flavors (short-chain). Butyrate methyl ester was the most important ester component, reaching a maximum content of 11,171.05 ng/g at 150 °C. Its fruit aroma not only masks the food‘s original aroma but also adds a sweet, fruity taste to deep-fried battered chicken breast strips [33].

Furans are Maillard reaction products and main aroma substances generated during heating [20]. 2-Pentylfuran is commonly associated with the oxidation and thermal degradation of polyunsaturated fatty acids, particularly linoleic acid, rather than with octanal. However, alkyl furan compounds have relatively high thresholds, so unlike other fat degradation products, they do not contribute significantly to battered chicken breast strips [34].

Pyrazines are formed by the condensation of α-aminoketones generated from amino acid degradation, contributing to baking, roasting, and nutty flavors [30]. Heat treatment increases the rate of amino acid-involved Maillard reactions, and during frying, amino acids and reducing sugars in the batter and meat undergo Maillard reactions that generate pyrazines. The amine peak putatively annotated as triethylenediamine was excluded from flavor interpretation as noted in Section 3.4.2.

#### 3.4.4. Relative Content Analysis

**Figure 5:**
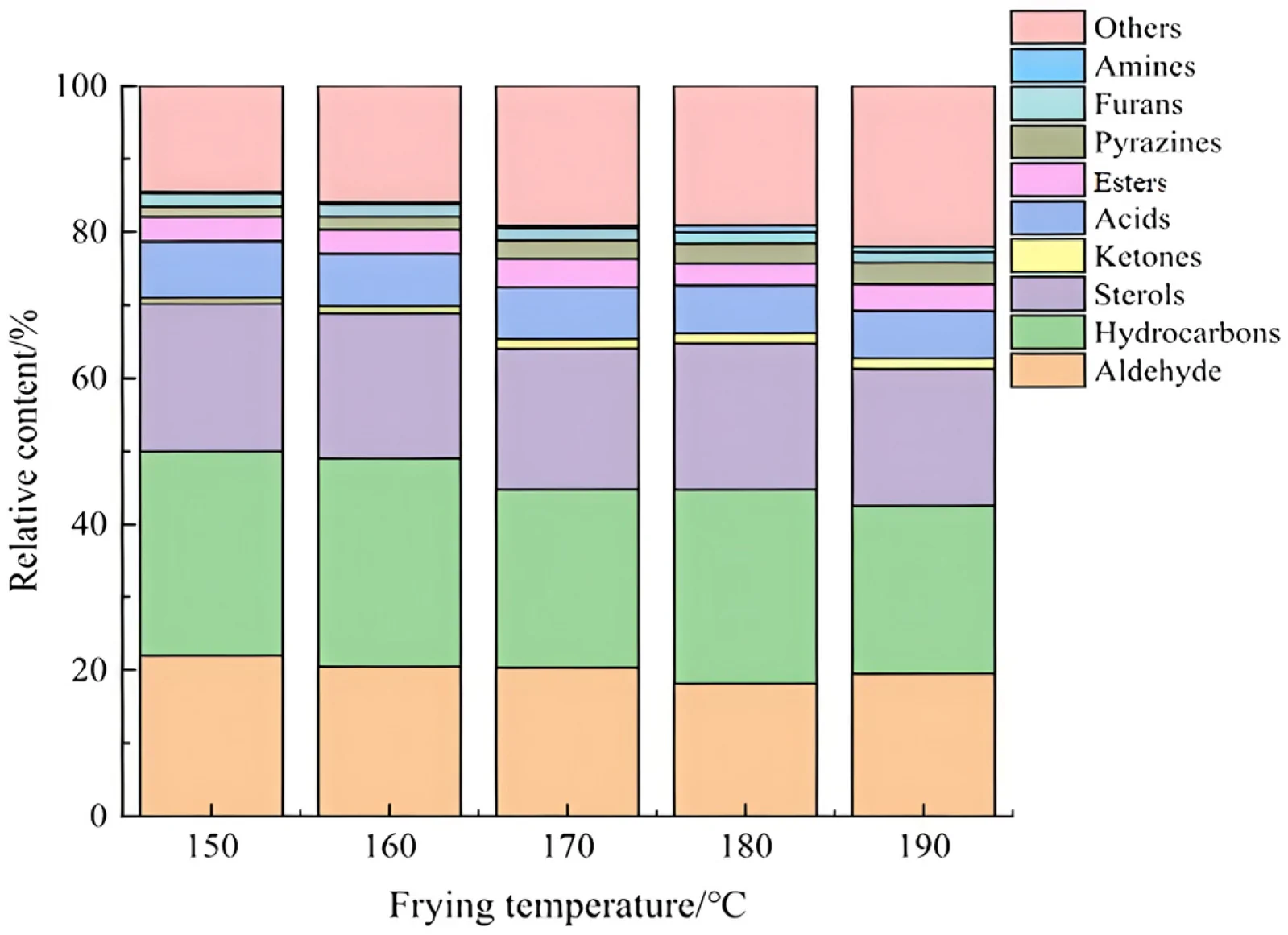
Relative contents of volatile components in deep-fried battered chicken breast strips at different frying temperatures.

The relative contents of volatile components in deep-fried chicken breast strips at different temperatures were derived from fingerprint signal intensities (Figure 5). Across temperatures, aldehydes accounted for 18.20–21.95%, hydrocarbons 23.01–27.99%, alcohols 18.77–20.31%, ketones 0.15–0.73%, acids 6.53–7.69%, esters 3.00–3.87%, pyrazines 1.47–3.87%, furans 1.47–3.00%, amines 0.24–0.98%, and unidentified substances 14.54–21.99%. With increasing frying temperature, the relative contents of pyrazines, amines, and ketones gradually increased, whereas those of aldehydes, hydrocarbons, alcohols, acids, esters, furans, and unidentified compounds gradually declined. This divergence can be attributed to the combined effects of two competing reaction pathways. On the one hand, higher temperatures promote the Maillard reaction between amino acids and reducing sugars, leading to the enhanced generation of pyrazines and other nitrogen-containing heterocyclic compounds as secondary reaction products. On the other hand, aldehydes and other thermally labile volatiles, despite being continuously generated via lipid oxidation and Strecker degradation, are subject to accelerated evaporation and thermal decomposition at elevated frying temperatures, resulting in a net decrease in their relative abundance [35].

#### 3.4.5. PCA

**Figure 6:**
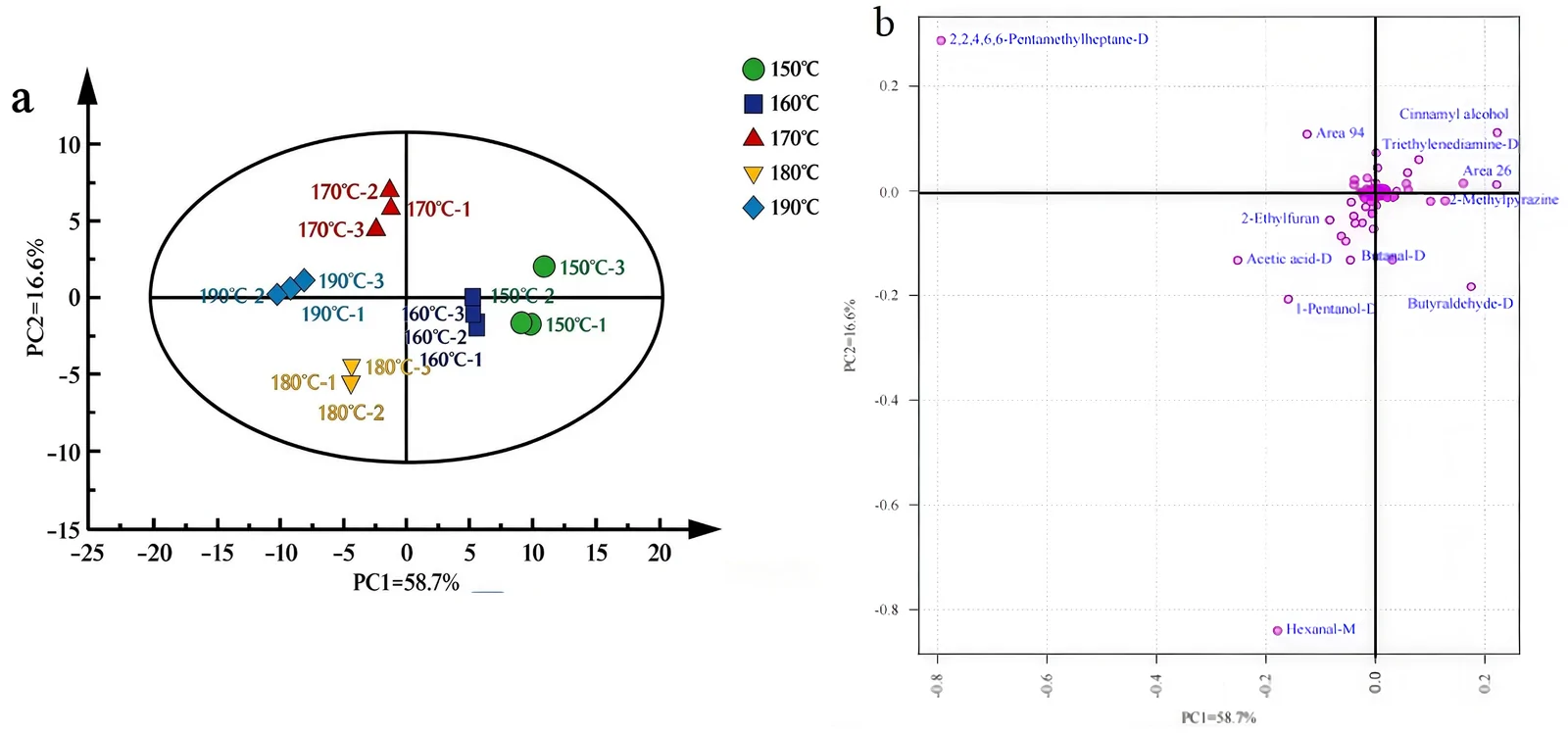
Principal component analysis (PCA) score plot of volatile compounds in batter-coated chicken strips fried at different temperatures. (**a**) PCA score plot of samples; (**b**) PCA loading plot of volatile compounds.

PCA reduces data dimensionality by identifying principal components (directions of maximum variance). PCA was performed using the relative contents of aroma substances (Figure 6a). The first two principal components (PC1 and PC2) accounted for 75.3% of the total variance (PC1: 58.7%, PC2: 16.6%). Samples at 150–190 °C were clearly separated with no cross-overlap, indicating significant differences in the overall aroma profiles across frying temperatures [36]. The loading plot (Figure 6b) showed that 2,2,4,6,6-pentamethylheptane-D, acetic acid-D, 2-ethylfuran, 2-methylpyrazine, and cinnamyl alcohol were the main contributors to PC1, whereas hexanal-M, 1-pentanol-D, butanal-D, triethylenediamine-D, and butyraldehyde-D dominated PC2. These results suggest that pyrazines, furans, and certain aldehydes are the key discriminators among different frying temperatures, reflecting the temperature-dependent balance between Maillard reaction products (e.g., 2-methylpyrazine) and lipid oxidation-derived aldehydes (e.g., hexanal) [36].

#### 3.4.6. OPLS-DA

**Figure 7:**
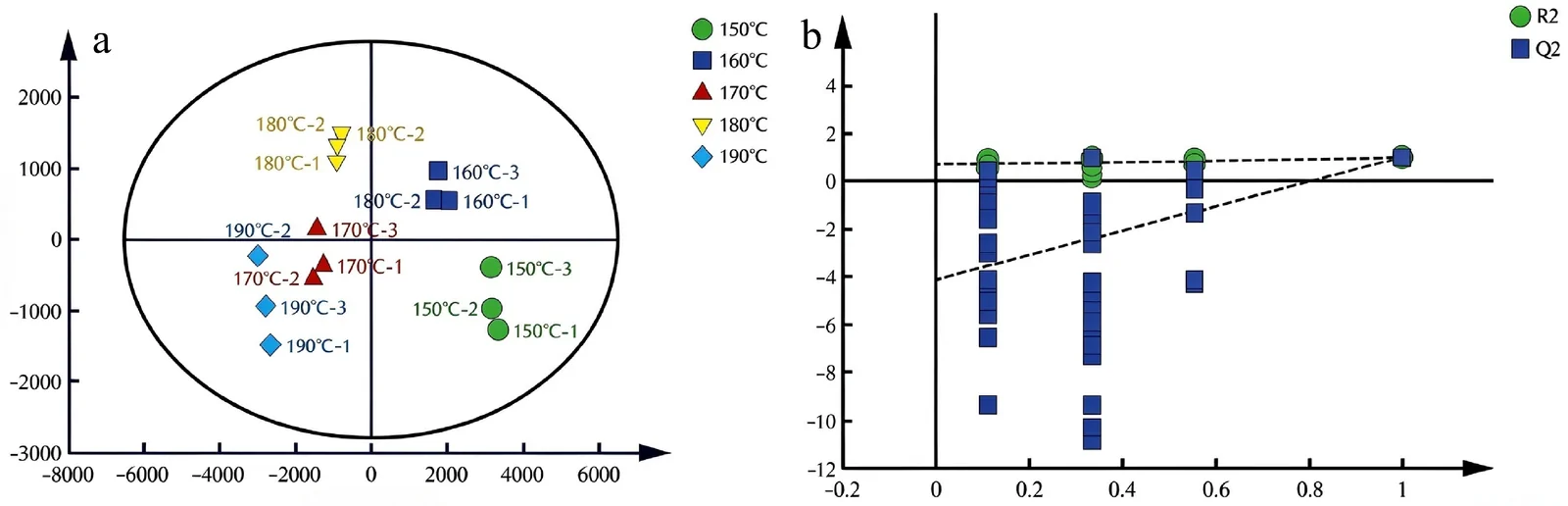
Scatter plot of OPLS-DA model scores for volatile components in fried chicken breasts at different deep-frying temperatures (**a**) and permutation test results of the OPLS-DA model (**b**).

Usually, PCA cannot fully explain the exact distances between samples. OPLS-DA concentrates classification information onto a single principal component, filtering out noise unrelated to group separation, thereby improving model interpretability and discriminatory power. To achieve more accurate results, supervised OPLS-DA was employed. After removing interfering factors, the model maximized intergroup differences. The sample distributions in the OPLS-DA score plot (Figure 7a) were similar to those from PCA. As shown in Table 4, the OPLS-DA model parameters were R^2^X = 0.992, R^2^Y = 0.924, and Q^2^ = 0.726, all close to 1, indicating a highly reliable model. The OPLS-DA results revealed significant differences in volatile component characteristics among groups, demonstrating that volatile compounds in deep-fried battered chicken breasts changed systematically with frying temperature.

After 200 permutation tests, the validity of the OPLS-DA model was further assessed to exclude overfitting. The Q^2^ values from the permuted models were all lower than the original Q^2^ value, and the Q^2^ regression line intersected the negative half of the vertical axis, indicating no overfitting and confirming the model’s robustness (Figure 7b).

#### 3.4.7. VIP Analysis

Based on the variable importance in projection (VIP) values predicted by the OPLS-DA model, we investigated the differentially characterized volatile compounds of five groups of deep-fried battered chicken breast strips, and the results are shown in Table 5. Using VIP > 1 as the screening condition, 11 compounds were statistically selected, among which 10 were retained as flavor-relevant differential volatile compounds, including four alcohols (cinnamyl alcohol, 1-pentanol-D, 3-methylbutan-1-ol-M), three aldehydes (hexanal-M, butyraldehyde-M, (Z)-4-decenal), one acid (acetic acid-D), one pyrazine (2-methylpyrazine), and one alkane (2,2,4,6,6-pentamethylheptane-D). Triethylenediamine was also statistically selected (VIP > 1) and is listed in Table 5 for completeness; however, it was excluded from the final flavor discussion because its identity could not be confirmed with an authentic standard, and its known industrial origin as a potential food-contact migrant precludes its interpretation as a frying-derived flavor compound (see Section 3.4.2).

**Figure 8:**
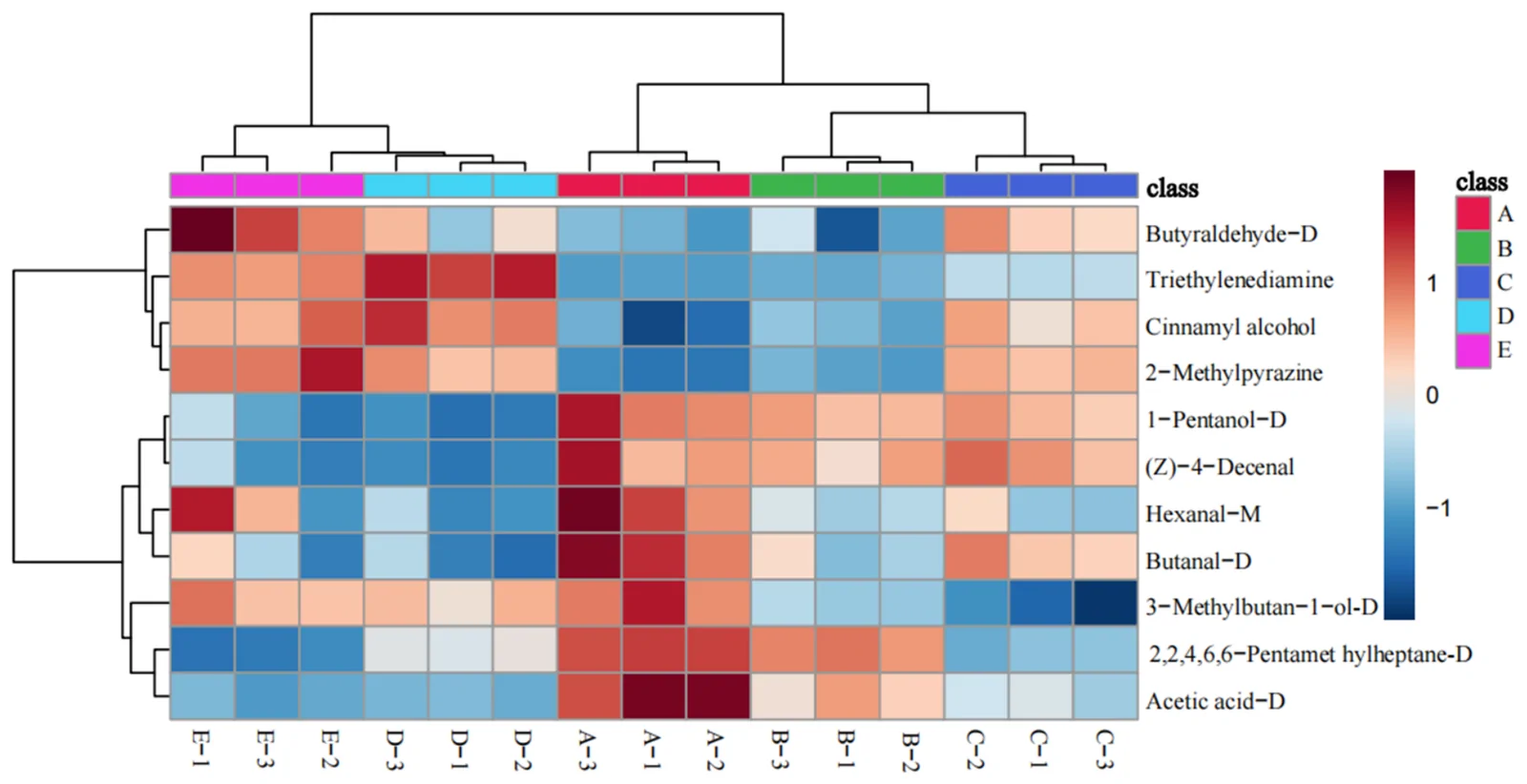
Hierarchical cluster analysis heatmap of volatile components in deep-fried chicken breast strips at different frying temperatures. A, B, C, D and E represent samples fried at 150 °C, 160 °C, 170 °C, 180 °C and 190 °C, respectively. Triethylenediamine and (Z)-4-decenal are included in the heatmap for data completeness; however, triethylenediamine was excluded from the flavor interpretation due to its industrial origin (Section 3.4.2), and (Z)-4-decenal was excluded from the OAV calculation due to the lack of a verified odor threshold (Section 3.4.8).

To better evaluate the differences in characteristic volatile compounds in deep-fried chicken breast strips at different deep-frying temperatures, hierarchical clustering analysis was conducted on the relative contents of 11 selected volatile compounds, which are displayed in the form of heatmaps (Figure 8). The results revealed that, when fried at 150 °C, mainly 1-pentanol-D, (Z)-4-decenal, hexanal-M, butanal-D, 3-methylbutan-1-ol, 2,2,4,6,6-pentamethylheptane-D, and acetic acid-D, these chemicals may have low boiling points and be easily volatilized at high temperatures. Moreover, higher deep-frying temperatures might cause a series of complex chemical reactions, leading to the decomposition, transformation, or loss of volatile compounds, thereby reducing their content [37]. When the deep-frying temperature was above 170 °C, the contents of butyraldehyde-D, triethylenediamine, cinnamyl alcohol, and 2-methyl pyrazine gradually increased, possibly because of a series of thermochemical reactions in the fat, protein, and carbohydrates of the ingredients when fried at high temperatures, accelerating the oxidation of the fats in the ingredients or the reactions between sugars and amino acids. Pyrazine is the most common aroma in cooked meat products, which to some extent reflects the Maillard reaction process [38].

Triethylenediamine (entry 2) exhibited a VIP value > 1 and is listed for statistical completeness. However, it was excluded from the subsequent flavor discussion and OAV analysis because it is likely a food-contact migrant rather than a frying-derived flavor compound (see Section 3.4.7).

#### 3.4.8. Odor Activity Values of Differential Volatiles

Odor activity values (OAVs) were calculated as the ratio of the semi-quantitative concentration of each compound to its corresponding odor threshold, with both expressed on a compatible basis. Numerous compounds exhibited OAV > 1 across all temperatures from 150 °C to 190 °C, indicating their persistent contributions to the overall aroma profile. Table 6 presents the key OAV > 1 compounds that were either VIP-selected or representative of major chemical classes. When further intersected with the VIP > 1 differential volatiles, three compounds were identified as both sensorially active (OAV > 1 across all temperatures) and statistically critical for sample discrimination (VIP > 1): hexanal-M, butyraldehyde-M, and 3-methylbutan-1-ol-M. Among these, hexanal-M and butyraldehyde-M, typical products of lipid oxidation and Strecker degradation, exhibited the highest OAVs (>10^5^ throughout the temperature range), whereas 3-methylbutan-1-ol-M showed lower OAVs (34–49), reflecting differences in the metabolic pathway of branched-chain amino acids. (Z)-4-Decenal was also VIP-selected; however, its odor threshold could not be traced to a reliable primary literature source under our experimental conditions, and its OAV was therefore not calculated. It is discussed only in terms of its relative abundance (Table 3). Taken together, these three compounds are recommended as priority markers for differentiating thermal processing temperatures and for aroma regulation in heat-treated products. It should be noted that all OAVs reported here are based on semi-quantitative concentrations and should therefore be regarded as relative indicators of aroma contribution rather than as precise absolute values.

Only compounds with OAV > 1 at all temperatures that are either VIP-selected or representative of major chemical classes are listed.

#### 3.4.9. Correlation Analysis

Pearson correlation analysis was performed on the relative peak volumes of the selected volatile compounds against sensory scores and total fat content across all 15 samples (*n* = 15). The sensory score was strongly negatively correlated with the content of 3-methylbutan-1-ol (isoamyl alcohol) (r = −0.742, *p* = 0.0015). The precursor origin of this compound (meat vs. batter) cannot be resolved from the present GC-IMS data alone. Both chicken muscle proteins and wheat flour proteins are leucine-rich, and both matrices undergo thermal degradation during frying [14]. In the present study, higher levels of isoamyl alcohol in the samples coincided with lower sensory scores. One possible interpretation is that elevated isoamyl alcohol content may be associated with more intense frying conditions (e.g., higher oil temperatures or prolonged frying times), which could lead to overcooked or burnt attributes and a deterioration in aroma. However, further studies are needed to establish a causal relationship between frying parameters and isoamyl alcohol formation.

**Figure 9:**
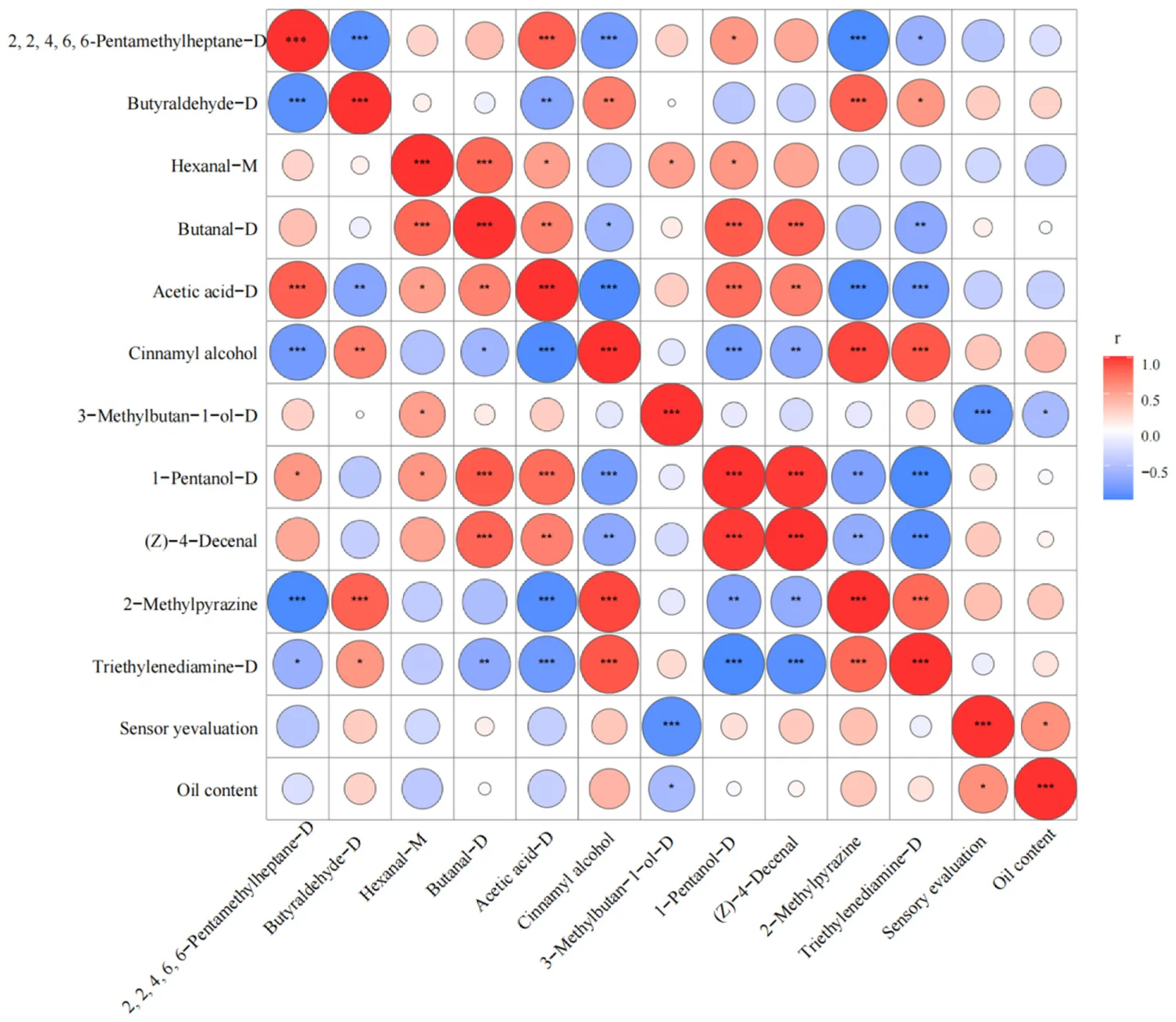
Pearson correlation analysis between the 11 selected volatile aroma compounds and sensory evaluation/total fat content in deep-fried chicken breasts at different temperatures (*n* = 15). * indicates a significant correlation (*p* < 0.05), ** indicates an extremely significant correlation (*p* < 0.01), and *** indicates an ultra-extremely significant correlation (*p* < 0.001). Triethylenediamine and (Z)-4-decenal are shown for data transparency but were not retained for the final flavor interpretation (see Section 3.4.2 and Section 3.4.8).

Conversely, cinnamyl alcohol (r = 0.784, *p* = 0.0006), 2-methylpyrazine (r = 0.799, *p* = 0.0004), and butyraldehyde-D (r = 0.591, *p* = 0.0203) were positively correlated with the sensory score, and cinnamyl alcohol (r = 0.675, *p* = 0.0058) and 2-methylpyrazine (r = 0.615, *p* = 0.0149) were also positively correlated with the total fat content. These findings suggest that the deep-frying temperature is crucial for forming the distinctive aroma of volatile components in battered chicken breasts. The unique aroma contributed by these compounds enhanced the overall flavor of the deep-fried chicken breasts (Figure 9). Triethylenediamine and (Z)-4-decenal were not interpreted as flavor-relevant in this analysis.

## 4. Conclusions

This study investigated the effects of deep-frying temperature (150–190 °C) on the quality and volatile flavor profiles of batter-coated chicken breast strips using an E-nose and GC–IMS. Sensory scores and oil content peaked at 170 °C for 170 s (83.0 ± 0.82 and 49.8%, respectively) and declined thereafter, while textural hardness increased sharply above 170 °C, indicating excessive dehydration and protein aggregation. Sixty volatile compounds were identified across all samples. Multivariate analysis (PCA and OPLS-DA) clearly discriminated samples by temperature, with 10 key differential compounds screened via VIP > 1. Correlation analysis revealed that cinnamyl alcohol, 2-methylpyrazine, and butyraldehyde-D were positively correlated with sensory attributes, whereas 3-methylbutan-1-ol was negatively correlated. OAV analysis further identified (Z)-4-decenal, hexanal-M, butyraldehyde-M, and 3-methylbutan-1-ol-M as the predominant aroma-active compounds. These findings suggest that frying temperature critically modulates the balance between lipid oxidation and Maillard reactions, thereby shaping the volatile profile and sensory quality. The integrated E-nose/GC–IMS approach proved effective for comprehensive flavor characterization, providing a theoretical basis for optimizing chicken frying processes. It should be noted that the core temperature of the chicken strips was not measured in this study; although all samples had identical dimensions and frying time, the core temperature was governed primarily by the oil temperature. Future studies should record core temperature profiles to further support the practical applicability of these findings. Future research should also explore the interplay between flavor development and nutritional changes during deep frying.

## Figures and Tables

**Table 1:** Sensors and corresponding performance of the E-nose.

Serial Number	Sensor Name	Response Substance
1	S1	Short-chain alkanes
2	S2	Carbon-containing substances, etc.
3	S3	Hydrogen
4	S4	Sulfide
5	S5	Nitrogen-containing substances
6	S6	Aldehydes and ketones

**Table 2:** Instrumental texture profile parameters of batter-coated chicken strips fried at different temperatures.

Temperature (°C)	Hardness (N)	Springiness (mm)	Gumminess (N)	Chewiness (mJ)
150	11.22 ± 0.97 ^c^	3.77 ± 0.97 ^c^	0.61 ± 0.17 ^d^	2.40 ± 0.30 ^b^
160	13.00 ± 4.20 ^bc^	4.82 ± 0.31 ^c^	1.42 ± 0.21 ^c^	2.53 ± 0.32 ^b^
170	18.20 ± 1.52 ^bc^	6.45 ± 0.42 ^bc^	1.65 ± 0.25 ^c^	2.72 ± 0.43 ^b^
180	30.7 ± 6.56 ^ab^	7.97 ± 1.09 ^b^	1.96 ± 0.27 ^b^	4.65 ± 0.35 ^a^
190	35.31 ± 5.02 ^a^	9.31 ± 1.09 ^a^	2.80 ± 0.25 ^a^	4.74 ± 0.23 ^a^

Springiness is expressed as instrumental recovery (mm) measured by TPA. Different superscript letters (a–d) within the same column indicate significant differences (*p* < 0.05).

**Table 3:** Identification of volatile compounds in deep-fried chicken breast strips at different temperatures.

Compound Category	No.	Name	Formula	RI	Rt (s)	Dt (ms)	Aroma Description	Peak Volume				
								150 °C	160 °C	170 °C	180 °C	190 °C
Aldehydes	1	(E)-2-Hexenal	C_6_H_10_O	1220.4	769.436	1.17901	Cheese, Fruity	317.31 ± 34.07 ^a^	317.31 ± 10.13 ^a^	337.97 ± 7.76 ^a^	252.26 ± 7.72 ^b^	269.62 ± 10.09 ^b^
	2	(Z)-4-Decenal	C_10_H_18_O	1190.2	670.358	1.34101	Citrus, Herbal	1955.53 ± 141.40 ^a^	1848.91 ± 71.54 ^a^	1913.27 ± 73.63 ^a^	1444.26 ± 21.87 ^b^	1523.09 ± 117.61 ^b^
	3	3-Methylbutanal	C_5_H_10_O	883.2	279.135	1.195	--	242.12 ± 23.63 ^abc^	226.01 ± 8.96 ^bc^	258.84 ± 5.93 ^a^	213.98 ± 13.92 ^c^	245.38 ± 16.35 ^ab^
	4	Hexanal-M	C_6_H_12_O	1095.8	458.57	1.27731	Nutty	6567.48 ± 604.12 ^a^	4803.07 ± 231.17 ^b^	4791.36 ± 538.09 ^b^	4241.88 ± 474.37 ^b^	5524.63 ± 1382.04 ^ab^
	5	Hexanal-D	C_6_H_12_O	1090.8	452.815	1.55179	Herbal, Oil	1062.65 ± 33.28 ^ab^	1083.90 ± 20.55 ^a^	1013.96 ± 20.14 ^bc^	1074.94 ± 11.61 ^ab^	969.02 ± 53.79 ^c^
	6	Nonanal-M	C_9_H_18_O	1396.6	1322.834	1.48674	Floral, Fruity	4296.18 ± 95.47 ^a^	3491.48 ± 97.45 ^bc^	4123.06 ± 71.42 ^ab^	3798.44 ± 114.25 ^c^	3912.82 ± 159.06 ^bc^
	7	Nonanal-D	C_9_H_18_O	1398	1327.004	1.93503	Floral, Fruity	604.80 ± 37.45 ^ab^	516.23 ± 34.57 ^c^	647.89 ± 50.80 ^a^	465.24 ± 26.80 ^c^	525.68 ± 44.68 ^bc^
	8	Octanal-D	C_8_H_16_O	1294.4	1016.3	1.41786	Fresh, Oil	1034.58 ± 88.98 ^a^	949.54 ± 23.49 ^a^	922.31 ± 66.47 ^ab^	722.23 ± 6.05 ^c^	813.43 ± 76.01 ^bc^
	9	Octanal-M	C_8_H_16_O	1292.4	1009.713	1.8234	Oil	98.76 ± 6.43 ^a^	77.38 ± 2.69 ^b^	80.77 ± 7.48 ^b^	69.21 ± 5.89 ^b^	78.02 ± 12.11 ^b^
	10	Propionaldehyde-M	C_3_H_6_O	779.8	219.82	1.06179	Nutty	81.55 ± 5.49 ^a^	62.93 ± 6.68 ^b^	81.75 ± 4.57 ^a^	30.86 ± 1.74 ^c^	61.96 ± 8.43 ^b^
	11	Propionaldehyde-D	C_3_H_6_O	798.3	230.429	1.13297	Nutty	61.64 ± 3.76 ^b^	51.93 ± 2.28 ^c^	66.68 ± 2.81 ^ab^	62.43 ± 4.49 ^b^	74.23 ± 6.70 ^a^
	12	Valeraldehyde	C_5_H_10_O	992.1	341.583	1.19485	Nutty	226.78 ± 16.89 ^a^	220.18 ± 7.70 ^a^	195.88 ± 20.76 ^a^	209.38 ± 1.70 ^a^	202.51 ± 17.79 ^a^
	13	Butyraldehyde-M	C_4_H_8_O	833.2	250.442	1.14009	Nutty	11,586.16 ± 78.89 ^c^	11,548.74 ± 341.06 ^c^	12,210.84 ± 153.49 ^ab^	11,992.23 ± 275.41 ^bc^	12,647.23 ± 266.22 ^a^
	14	Butyraldehyde-D	C_4_H_8_O	839.1	253.817	1.28551	Nutty	968.41 ± 21.92 ^e^	1074.31 ± 7.38 ^d^	1265.70 ± 8.35 ^b^	1237.58 ± 11.04 ^c^	1297.66 ± 10.56 ^a^
Hydrocarbons	1	Alpha-pinene	C_10_H_16_	1023.1	374.945	1.22801	Spicy	502.20 ± 19.77 ^a^	475.81 ± 5.15 ^b^	425.05 ± 12.53 ^c^	470.93 ± 2.53 ^b^	433.23 ± 12.53 ^c^
	2	2,2,4,6,6-Pentamethylheptane-M	C_12_H_26_	954.4	319.94	1.14116	--	22,523.11 ± 105.16 ^a^	21,780.92 ± 217.84 ^b^	18,814.30 ± 210.78 ^d^	200,400.39 ± 153.70 ^c^	17,773.62 ± 218.32 ^e^
	3	2,2,4,6,6-Pentamethylheptane-D	C_12_H_26_	957.5	321.753	1.41105	--	70.03 ± 1.04 ^d^	75.11 ± 7.95 ^d^	155.87 ± 12.39 ^b^	126.96 ± 4.04 ^c^	202.85 ± 12.69 ^a^
	4	Decalin	C_10_H_18_	1153.4	587.408	1.35527	--	46.67 ± 1.37 ^d^	54.69 ± 2.83 ^d^	86.83 ± 1.66 ^c^	124.90 ± 8.55 ^b^	161.61 ± 3.43 ^a^
	5	Myrcene	C_10_H_16_	1160	602.175	1.27358	Lemon	77.06 ± 9.95 ^a^	63.49 ± 1.32 ^b^	64.34 ± 0.86 ^b^	41.85 ± 1.05 ^c^	44.72 ± 9.35 ^c^
	6	p-Xylene	C_8_H_10_	1147.7	574.486	1.07263	--	29.31 ± 1.48 ^e^	46.31 ± 7.65 ^d^	99.84 ± 4.55 ^c^	124.87 ± 1.94 ^b^	188.00 ± 3.32 ^a^
	7	Terpinolene	C_10_H_16_	1291.1	1005.253	1.20553	Lemon, Floral	105.12 ± 9.68 ^d^	126.44 ± 11.26 ^c^	160.26 ± 9.71 ^b^	164.31 ± 4.38 ^ab^	178.06 ± 6.92 ^a^
	8	Undecane-M	C_11_H_24_	1104.1	475.864	1.09502	--	804.04 ± 19.01 ^a^	771.67 ± 11.62 ^a^	756.74 ± 5.48 ^a^	784.53 ± 15.88 ^a^	777.87 ± 41.68 ^a^
	9	Undecane-D	C_11_H_24_	1090.5	452.412	1.36868	--	232.32 ± 1.83 ^c^	223.41 ± 11.91 ^c^	352.86 ± 19.68 ^b^	370.89 ± 14.17 ^b^	485.90 ± 11.24 ^a^
Alcohols	1	1-Pentanol-M	C_5_H_12_O	1258.3	895.924	1.25768	Oil	3012.74 ± 214.04 ^a^	2719.45 ± 72.79 ^a^	2714.07 ± 118.74 ^a^	1763.01 ± 87.14 ^b^	1989.90 ± 278.00 ^b^
	2	1-Pentanol-D	C_5_H_12_O	1258	894.974	1.51785	Oil	463.73 ± 69.34 ^a^	368.43 ± 21.10 ^b^	368.64 ± 21.72 ^b^	159.02 ± 18.17 ^c^	202.21 ± 48.82 ^c^
	3	2-Butanol-M	C_4_H_10_O	1024.1	376.154	1.15177	Fruity	1561.30 ± 50.22 ^a^	1454.44 ± 15.41 ^b^	1370.04 ± 18.01 ^c^	1439.819.14 ^b^	1289.60 ± 12.01 ^d^
	4	2-Butanol-D	C_4_H_10_O	1022.8	374.643	1.32415	Fruity	957.63 ± 11.58 ^a^	820.33 ± 5.54 ^b^	788.71 ± 30.52 ^b^	911.57 ± 14.87 ^a^	779.54 ± 45.34 ^b^
	5	2-Methylpropanol-M	C_4_H_10_O	1098.6	463.545	1.17171	--	1660.76 ± 79.98 ^a^	1673.04 ± 15.10 ^a^	1655.53 ± 36.13 ^a^	1764.30 ± 33.39 ^a^	1670.18 ± 87.91 ^a^
	6	2-Methylpropanol-M	C_4_H_10_O	1098.7	463.823	1.36768	--	623.86 ± 27.76 ^b^	522.38 ± 8.34 ^d^	492.95 ± 6.13 ^d^	574.02 ± 8.23 ^c^	555.22 ± 19.25 ^c^
	7	3-Heptanol	C_7_H_16_O	889.4	282.666	1.33518	Herbal	620.22 ± 36.04 ^a^	486.56 ± 23.76 ^b^	564.96 ± 12.27 ^ab^	397.81 ± 18.74 ^c^	600.38 ± 74.10 ^a^
	8	3-Methylbutan-1-ol-M	C_5_H_12_O	1210.8	737.152	1.24155	Fruity, Wine	2672.16 ± 85.32 ^a^	2321.42 ± 26.71 ^c^	2111.84 ± 84.90 ^d^	2517.30 ± 54.75 ^b^	2568.97 ± 70.69 ^ab^
	9	3-Methylbutan-1-ol-D	C_5_H_12_O	1210.4	1210.4	1.49286	Fruity, Wine	562.34 ± 28.13 ^a^	431.85 ± 2.87 ^c^	363.22 ± 21.84 ^d^	512.68 ± 4.67 ^b^	523.77 ± 31.10 ^ab^
	10	Butanol-M	C_4_H_10_O	1143.7	565.502	1.18152	Fruity, Wine	2023.93 ± 20.74 ^a^	1903.32 ± 30.04 ^b^	1831.15 ± 39.98 ^c^	1936.37 ± 27.33 ^b^	1823.57 ± 33.16 ^c^
	11	Butanol-D	C_4_H_10_O	1143.8	565.521	1.38631	Wine	390.10 ± 29.52 ^a^	330.11 ± 12.67 ^b^	273.85 ± 9.57 ^c^	329.68 ± 12.64 ^b^	282.10 ± 13.42 ^c^
	12	Cinnamyl alcohol	C_9_H_10_O	1305	1048.204	1.07512	Spicy, Floral	1733.38 ± 271.00 ^c^	2058.06 ± 87.36 ^c^	2733.77 ± 175.08 ^b^	3113.30 ± 192.23 ^a^	2932.43 ± 178.52 ^ab^
	13	Ethanol	C_2_H_6_O	902.6	290.226	1.0567	Wine	854.34 ± 11.69 ^a^	848.83 ± 21.35 ^a^	710.60 ± 8.13 ^b^	741.06 ± 10.41 ^b^	626.54 ± 23.26 ^c^
Ketones	1	Methyl heptenone	C_8_H_14_O	1340.2	1153.773	1.17628	Fruity	90.73 ± 7.06 ^a^	72.66 ± 3.94 ^b^	60.71 ± 4.37 ^c^	67.01 ± 3.22 ^bc^	64.61 ± 7.73 ^bc^
	2	1-Hydroxy-2-propanone acetate	C_5_H_8_O_3_	1475.7	1560.212	1.1008	Fruity, Nutty	166.88 ± 33.10 ^c^	184.22 ± 2.73 ^bc^	213.17 ± 13.39 ^ab^	214.59 ± 11.05 ^ab^	220.05 ± 7.53 ^a^
	3	2-Propanone, 1-Hydroxy-	C_3_H_6_O_2_	1305.3	1049.315	1.23131	Caramel	123.91 ± 41.64 ^c^	190.85 ± 16.12 ^c^	391.87 ± 55.13 ^b^	498.55 ± 66.40 ^a^	424.77 ± 62.73 ^ab^
	4	3-Hydroxy-2-Butanone	C_4_H_8_O_2_	1291	1004.872	1.32846	Creamy	144.25 ± 14.97 ^c^	185.28 ± 5.55 ^c^	321.44 ± 18.20 ^ab^	292.05 ± 25.87 ^b^	368.19 ± 38.86 ^a^
	5	Cyclopentanone	C_5_H_8_O	1205.8	720.652	1.09151	Minty	108.27 ± 3.31 ^c^	113.25 ± 6.91 ^bc^	137.88 ± 0.56 ^a^	127.45 ± 15.51 ^ab^	140.25 ± 6.68 ^a^
Acids	1	2-Heptenoic acid	C_7_H_12_O_2_	1189.8	669.533	1.43771	Fruity	299.82 ± 28.96 ^a^	274.63 ± 16.27 ^a^	267.09 ± 11.65 ^a^	222.80 ± 9.50 ^b^	222.08 ± 12.52 ^b^
	2	2-Methylbutanoic acid	C_5_H_10_O_2_	860.3	265.969	1.19812	Spicy, Cheese	960.62 ± 12.86 ^e^	1105.48 ± 30.24 ^d^	1607.54 ± 27.81 ^b^	1446.09 ± 16.77 ^c^	1689.08 ± 40.76 ^a^
	3	2-Methylpentanoic acid	C_6_H_12_O_2_	1037.2	391.131	1.25934	Cheese	303.62 ± 8.89 ^a^	263.24 ± 6.06 ^b^	248.93 ± 17.73 ^b^	263.41 ± 4.54 ^b^	250.65 ± 10.44 ^b^
	4	Acetic acid-M	C_2_H_4_O_2_	1506.4	1652.121	1.05922	Spicy, Fruity	4899.38 ± 241.75 ^a^	4091.69 ± 196.75 ^b^	3670.22 ± 139.01 ^c^	3356.20 ± 37.17 ^cd^	3297.98 ± 82.99 ^d^
	5	Acetic acid-D	C_2_H_4_O_2_	1489.7	1.16087	1.16087	Spicy, Fruity	176.15 ± 27.49 ^ab^	170.24 ± 22.78 ^ab^	186.52 ± 3.58 ^a^	140.93 ± 0.91 ^b^	148.57 ± 17.56 ^b^
Esters	1	Benzyl butanoate	C_11_H_14_O_2_	1333.8	1134.524	1.43052	Fruity	290.51 ± 60.75 ^a^	200.13 ± 5.91 ^a^	188.08 ± 47.54 ^a^	199.45 ± 9.01 ^a^	260.65 ± 82.55 ^a^
	2	Butyl 2-propenoate-M	C_7_H_12_O_2_	1189.1	667.945	1.26405	--	446.48 ± 42.04 ^a^	429.44 ± 26.56 ^ab^	454.28 ± 16.44 ^a^	378.08 ± 30.01 ^b^	464.89 ± 16.95 ^a^
	3	Butyl 2-propenoate-D	C_7_H_12_O_2_	1190.1	670.237	1.68979	--	326.04 ± 49.06 ^a^	277.68 ± 25.42 ^a^	316.13 ± 44.30 ^a^	165.14 ± 8.80 ^b^	193.48 ± 30.83 ^b^
	4	Isobutyl propanoate	C_7_H_14_O_2_	865.8	269.156	1.27725	Banana, Fruity	241.09 ± 4.72 ^e^	337.95 ± 23.57 ^d^	730.99 ± 40.00 ^b^	600.71 ± 10.88 ^c^	824.49 ± 9.72 ^a^
	5	Methyl 3-Methylbutanoate	C_6_H_12_O_2_	1014	364.524	1.18024	Apple, Fruity	46.67 ± 9.30 ^d^	41.75 ± 2.96 ^d^	96.61 ± 2.88 ^b^	83.00 ± 1.70 ^c^	116.51 ± 4.06 ^a^
	6	Methyl Anthranilate	C_8_H_9_NO_2_	1324.3	1106.115	1.25692	Fruity	339.09 ± 35.60 ^a^	338.23 ± 21.77 ^a^	328.52 ± 5.69 ^a^	185.89 ± 6.84 ^b^	175.62 ± 9.23 ^b^
	7	Methyl Butyrate	C_5_H_10_O_2_	991.2	341.067	1.41377	Incense	11,171.05 ± 65.91 ^b^	1006.64 ± 50.24 ^b^	1012.46 ± 74.57 ^b^	712.80 ± 56.67 ^c^	818.52 ± 148.10 ^c^
	8	Methyl Phenyl Acetate	C_9_H_10_O_2_	1182.8	653.668	1.22484	Honey	57.47 ± 4.21 ^e^	66.47 ± 1.65 ^d^	132.88 ± 6.07 ^c^	148.61 ± 4.93 ^b^	265.61 ± 4.23 ^a^
Pyrazine	1	2,5-Dimethylpyrazine	C_6_H_8_N_2_	1341.3	1157.238	1.11629	Nutty, Oil	106.30 ± 5.68 ^e^	173.71 ± 9.49 ^d^	299.82 ± 13.31 ^c^	366.07 ± 22.74 ^b^	494.41 ± 37.54 ^a^
	2	2-Methylpyrazine	C_5_H_6_N_2_	1291	1004.872	1.07329	Nutty	1159.67 ± 50.71 ^c^	1297.92 ± 44.44 ^c^	1842.50 ± 38.85 ^b^	1861.19 ± 75.33 ^b^	2080.75 ± 139.07 ^a^
Furan	1	2-Ethylfuran	C_6_H_8_O	989.1	339.887	1.30043	Nutty	2863.34 ± 101.48 ^a^	2702.98 ± 48.09 ^ab^	2564.69 ± 59.13 ^bc^	2421.66 ± 54.81 ^cd^	2323.40 ± 133.25 ^d^
	2	2-Pentylfuran	C_9_H_14_O	1233	811.513	1.25808	Caramel	60.08 ± 5.34 ^c^	75.93 ± 3.61 ^b^	84.97 ± 8.14 ^ab^	85.42 ± 4.80 ^ab^	89.07 ± 8.03 ^a^
Amines	1	N-nitrosodiethylamine	C_4_H_10_N_2_O	898.5	287.875	1.17576	--	49.80 ± 4.11 ^a^	46.92 ± 2.11 ^a^	47.94 ± 2.74 ^a^	46.90 ± 1.05 ^a^	52.37 ± 4.00 ^a^
	2	Triethylenediamine-M	C_6_H_12_N_2_	1499.5	1631.266	1.09161	--	160.18 ± 3.32 ^d^	195.38 ± 12.74 ^d^	318.50 ± 6.02 ^c^	764.18 ± 36.51 ^a^	604.56 ± 21.15 ^b^
	3	Triethylenediamine-D	C_6_H_12_N_2_	1506.2	1651.409	1.16234	--	279.95 ± 42.00 ^a^	210.56 ± 39.49 ^b^	168.91 ± 3.16 b^c^	140.01 ± 6.86 ^c^	157.22 ± 10.91 ^c^

Data are presented as mean ± SD. Different superscript letters in the same row indicate significant differences (*p* < 0.05, Duncan’s multiple range test).

**Table 4:** Parameters of the OPLS-DA model of volatile components in deep-fried battered chicken breast strips at different frying temperatures.

Data Origin	R^2^X (cum)	R^2^Y (cum)	Q^2^ (cum)
GC–IMS	0.992	0.924	0.726

**Table 5:** VIP values for differences in volatile components in deep-fried battered chicken breast strips at different deep-frying temperatures based on OPLS-DA.

No.	CAS	Aroma Substance	VIP	Aroma Descriptions
1	123-72-8	Butyraldehyde−D	1.39195	Nutty
2	280-57-9	Triethylenediamine	1.18458	--
3	104-54-1	Cinnamyl alcohol	2.51251	Floral, Honey
4	109-08-0	2-Methylpyrazine	1.25076	Nutty
5	71-41-0	1-Pentanol-D	2.31214	Oil, Fruity
6	21662-09-9	(Z)−4-Decenal	1.1712	Orange, Fruity, Floral, Oil
7	66-25-1	Hexanal−M	3.90121	Fruity, Oil
8	123-72-8	Butanal−D	1.21381	Nutty
9	123-51-3	3-Methylbutan-1-ol-D	1.3023	Oil, Banana, Wing
10	13475-82-6	2,2,4,6,6-Pentamethylheptane-D	5.46001	--
11	64-19-7	Acetic acid−D	2.20713	Acidic, Spicy, Fruity

**Table 6:** OAV of volatile compounds in deep-fried chicken breast strips at different temperatures.

Compound	Threshold (mg/kg)	150 °C	160 °C	170 °C	180 °C	190 °C
(E)-2-Hexenal	0.017	14.65	17	19.06	18.12	18.18
3-Methylbutanal	0.00015	1267	1373	1653	1747	1873
Hexanal-M	0.0045	1147	973.6	1019	1152	1406
Hexanal-D	0.0045	185.6	219.8	215.6	291.8	246.7
Nonanal-M	0.001	3377	3185	3947	4641	4481
Nonanal-D	0.001	475	471	620	568	602
Octanal-D	0.0007	1161	1237	1261	1261	1331
Octanal-M	0.0007	111.4	101.4	110	121.4	127.1
Propionaldehyde-M	0.0095	6.74	5.20	8.21	4	7.47
Propionaldehyde-D	0.0095	5.05	4.95	6.74	8	8.95
Valeraldehyde	0.012	14.83	16.75	15.58	21.33	19.33
Butyraldehyde-M	0.009	1012	1170	1299	1628	1627
Butyraldehyde-D	0.009	84.56	108.9	134.7	168	165.1
Alpha-pinene	0.006	65.83	72.33	67.83	95.83	82.67
Myrcene	0.013	4.69	4.46	4.77	3.92	3.92
p-Xylene	0.53	0.04	0.08	0.18	0.29	0.41
Terpinolene	0.2	0.42	0.58	0.77	1.01	1.02
1-Pentanol-M	4	0.59	0.62	0.65	0.54	0.57
1-Pentanol-D	4	0.09	0.08	0.09	0.05	0.06
2-Butanol-M	50	0.02	0.03	0.03	0.04	0.03
2-Butanol-D	50	0.02	0.02	0.02	0.02	0.02
2-Methylpropanol-M (first)	0.55	2.38	2.78	2.88	3.92	3.48
2-Methylpropanol-M (second)	0.55	0.89	0.87	0.9	1.28	1.16
3-Heptanol	0.24	2.03	1.85	2.25	2.03	2.87
3-Methylbutan-1-ol-M	0.25	8.4	8.47	8.09	12.31	11.77
3-Methylbutan-1-ol-D	0.25	1.77	1.58	1.45	2.51	2.4
Butanol-M	0.5	3.18	3.47	3.51	4.73	4.18
Butanol-D	0.5	0.61	0.6	0.52	0.81	0.65
Ethanol	100	0.01	0.01	0.01	0.01	0.01
Methyl heptenone	0.05	1.42	1.32	1.16	1.64	1.48
2-Propanone, 1-Hydroxy-	80	0	0	0	0.01	0.01
3-Hydroxy-2-Butanone	0.9	0.13	0.19	0.34	0.4	0.47
Cyclopentanone	9.3	0.01	0.01	0.01	0.02	0.02
Acetic acid-M	22	0.18	0.17	0.16	0.19	0.17
Acetic acid-D	22	0.01	0.01	0.01	0.01	0.01
Benzyl butanoate	0.376	0.61	0.49	0.48	0.65	0.8
Butyl 2-propenoate-M	0.009	39	43.56	48.33	51.33	59.11
Butyl 2-propenoate-D	0.009	28.44	28.11	33.67	22.44	24.67
Isobutyl propanoate	0.26	0.73	1.19	2.69	2.82	3.63
Methyl 3-methylbutanoate	0.0044	8.41	8.64	20.91	22.95	30.23
Methyl anthranilate	0.003	89	103	104.7	75.67	67
Methyl butyrate	0.059	148.8	15.56	16.42	14.76	15.9
2,5-Dimethylpyrazine	1.7	0.05	0.09	0.17	0.26	0.33
2-Methylpyrazine	60	0.02	0.02	0.03	0.04	0.04
2-Pentylfuran	0.006	7.83	11.5	13.5	17.33	17

## Data Availability

The original contributions presented in this study are included in the article. Further inquiries can be directed to the corresponding author.
